# Birth Characteristics and Bone Mineral Density and Content in Young Adults: The HUNT Study, Norway

**DOI:** 10.1007/s00223-025-01441-2

**Published:** 2025-10-22

**Authors:** H. T. Holltrø, T. I. L. Nilsen, B. Schei, J. Horn, K. Holvik, A. K. N. Daltveit, E. M. Dennison, N. C. Harvey, A. Langhammer, M. Hoff

**Affiliations:** 1 Department of Neuromedicine and Movement Science, Faculty of Medicine and Health SciencesNTNU, Norwegian University of Science and Technology, 7491 Trondheim, Norway; 2 Department of Rheumatology, St Olavs University Hospital, Trondheim, Norway; 3 Department of Public Health and Nursing, Faculty of Medicine and Health Sciences, NTNU – Norwegian University of Science and Technology, Trondheim, Norway; 4 Clinic of Emergency Medicine and Prehospital Care, St. Olav’s Hospital, Trondheim University Hospital, Trondheim, Norway; 5 Department of Obstetrics and Gynecology, St. Olav’s Hospital, Trondheim University Hospital, Trondheim, Norway; 6 Department of Public Health and Nursing, Faculty of Medicine and Health Sciences, HUNT Research Centre, NTNU – Norwegian University of Science and Technology, Levanger, Norway; 7 Department of Obstetrics and Gynecology, Levanger Hospital, Nord-Trøndelag Hospital Trust, Levanger, Norway; 8 Department of Physical Health and Ageing, Norwegian Institute of Public Health, Oslo, Norway; 9 Department of Global Public Health and Primary Care, University of Bergen, Bergen, Norway; 10 Department of Health Registry Research and Development, Norwegian Institute of Public Health, Bergen, Norway; 11 MRC Lifecourse Epidemiology Centre, University of Southampton, Southampton, UK; 12 NIHR Southampton Biomedical Research Centre, University of Southampton and University Hospital Southampton NHS Foundation Trust, Southampton, UK; 13 Victoria University of Wellington, Wellington, New Zealand; 14 Levanger Hospital, Nord-Trøndelag Hospital Trust, Levanger, Norway

**Keywords:** Birth weight, Bone mineral density, Bone mineral content, Dual-energy X-ray, Young adults

## Abstract

**Purpose:**

To examine the association between birth characteristics and bone mineral density (BMD) and bone mineral content (BMC) in young adults.

**Methods:**

Data from 3,174 participants aged 20–54 years from the 3rd (2006–2008) and 4th (2017–2019) HUNT Study surveys were linked to the Medical Birth Registry of Norway. BMD and BMC of femoral neck were measured using dual-energy X-ray absorptiometry (DXA). Linear regression estimated mean differences in BMD and BMC by birth characteristics, adjusting for sex, birth year, age at scan, maternal age, and maternal morbidity.

**Results:**

At bone densitometry, participants had a mean age of 34.2 years, with mean BMD of 0.971 g/cm^2^, and mean BMC of 5.398 g, at the femoral neck. A standard deviation (SD) increase in ponderal index (PI) and birth weight was associated with higher BMD of 0.024 g/cm^2^ (95% CI 0.006, 0.042) and 0.015 g/cm^2^ (95% CI 0.009, 0.022). Individuals born large for gestational age (LGA) had 0.023 g/cm^2^ (95% CI 0.007, 0.039) higher BMD than those born appropriate for gestational age (AGA), while low birth weight (LBW)(< 2.5 kg) was associated with − 0.028 g/cm^2^ (95% CI − 0.053, − 0.003) lower BMD.

For BMC, an SD increase in PI and birth weight was associated with 0.171 g (95% CI 0.048, 0.293) and 0.146 g (95% CI 0.112, 0.181) higher BMC, respectively. LGA had 0.206 g (95% CI 0.090, 0.313) higher BMC, while LBW was associated with − 0.298 g (95% CI − 0.469, − 0.127) lower BMC.

**Conclusion:**

Higher ponderal index, birth weight, and gestational age were positively associated with BMD and BMC in young adulthood.

**Supplementary Information:**

The online version contains supplementary material available at 10.1007/s00223-025-01441-2.

## Introduction

Adult bone mass may reflect both conditions in fetal life, throughout childhood and into young adulthood. Peak bone mass is typically reached in the third decade of life (1); women in their early twenties and men in their late twenties. There is a progressive loss of bone mass after this age, and peak bone mass is therefore important for the risk of fractures later in life [1, 2]. Measures of bone mass quantify the amount of minerals in the bone and may therefore reflect bone strength [3]. This can be measured either as bone mineral content (BMC), defined as the total amount of bone mineral in a specific skeletal site (grams), or bone mineral density (BMD), defined as bone mineral content per area (g/cm²). Understanding the factors that influence BMD and BMC from fetal life through the attainment of peak bone mass is essential for early intervention and prevention of osteoporosis.

A limited number of studies have examined the association between birth characteristics and bone health indicators in young adults. Two systematic literature reviews from 2010 and 2011 summarized the link between birth weight and bone mass in adulthood [4, 5] and indicated a positive association between birth weight and BMC, with less clear results for BMD. Since then, some smaller studies (*N* = 46–557) [6–12] have examined the impact of very low birth weight [6–8, 11], being born small for gestational age (SGA) [7, 8], and being born very preterm [9, 12] on adult bone health. In all seven studies, these birth characteristics were associated with a lower BMD.

Maternal vitamin D status during pregnancy has been shown to play a role in fetal bone development. Vitamin D deficiency in pregnant women, especially during late pregnancy, has been associated with suboptimal bone development in childhood [13] and lower PBM in early adulthood [14]. Seasonal variation in sunlight exposure, which affects maternal vitamin D levels, can therefore have implications for fetal bone health.

In this study, we aimed to examine the association between birth characteristics (including birth weight, birth weight for gestational age, and gestational length) and BMD and BMC in young adults within a large population study. By examining a diverse population that represents a wide range of birth characteristics, we had the opportunity to investigate a broader spectrum of birth characteristics, beyond those born with very low birth weight or very preterm. We hypothesize that being born preterm, with low birth weight, or SGA will result in lower BMD and BMC in adulthood. Finally, we investigated whether the season of birth affects adult BMD, as it could be an indicator of maternal vitamin D status.

## Material and Methods

### Study Population and Data Sources

We used data on participants in the third and fourth surveys of the population-based Trøndelag Health Study (HUNT) linked with information from the Medical Birth Registry of Norway (MBRN) using the unique national 11-digit personal identification number.

### Medical Birth Registry of Norway

Data regarding birth characteristics was obtained from the MBRN. Established in 1967, the MBRN is a mandatory national health registry that gathers details on all births reported by maternity units across Norway, including home births [15]. This registry includes the names and personal identification numbers of both the newborn and the parents, along with information about the mother’s health prior to and during pregnancy, as well as the mode of delivery and any complications related to the birth. Additionally, it records various health metrics in the newborn, including birth length, weight, head circumference at birth, and the Apgar score, among other factors.

### HUNT

**Fig. 1:**
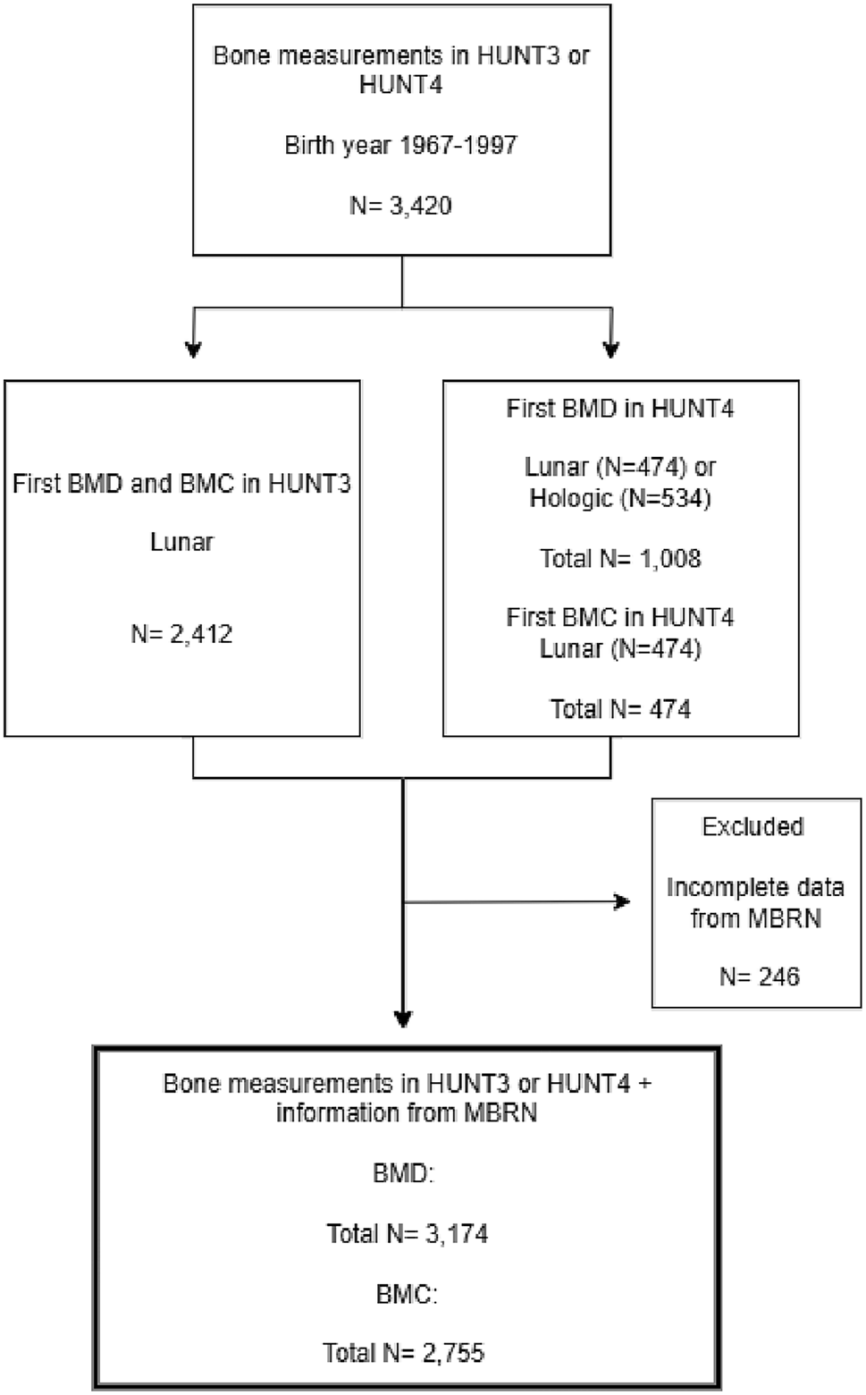
Flowchart of the included participants (*N* = number of participants)

The HUNT study is a longitudinal population-based health study that invited all inhabitants in the region of Nord-Trøndelag, Norway, aged 20 years and older to participate in repeated surveys [16]. So far, four surveys have been conducted: HUNT1 (1984–1986), HUNT2 (1995–1997), HUNT3 (2006–2008), and HUNT4 (2017–2019). In this study, we used data from HUNT3 and HUNT4. Participants completed comprehensive questionnaires and underwent a short clinical examination at the screening station. Additionally, a sample of participants was selected for bone densitometry. In HUNT3, participants were selected in two different ways: 1) a 10% random sample among all participants or as part of a 30% random sample from specific female birth cohorts 2) a sample reporting respiratory symptoms, diagnosis or use of medication for obstructive lung diseases and selected for the HUNT Lung Study [16]. The higher proportion of participants with pulmonary symptoms in HUNT3 was addressed in a sensitivity analysis to evaluate potential selection bias (Supplement). Participants invited for spirometry measurements were also asked to undergo bone densitometry measurements [17]. In HUNT4, individuals with bone densitometry in HUNT2 and/or HUNT3, still living in the region, were invited to new bone densitometry. In our study, we included all men and women with their first bone densitometry measurements from HUNT3 or HUNT4, born between 1967 and 1997, and with available birth characteristic information from the MBRN. This gave a total of 3,174 participants (1,900 women and 1,274 men) in this study (Fig. 1).

### Exposure: Birth Characteristics

Birth weight: Analyzed as a continuous variable/interval scale. The interval scale is defined as follows: less than 2.5 kg (low birth weight, LBW) [18]; 2.5–2.9 kg; 3.0–3.4 kg; 3.5–3.9 kg (reference group, representing the average birth weight in Norway [19]); 4.0–4.4 kg and 4.5 kg or more (high birth weight, HBW)).

Ponderal index (PI): A measure that relates an individual’s weight to their height, using height cubed (PI = weight in kg / height in m^3^). PI was analyzed as a continuous variable/interval scale. The interval scale for PI is defined as follows: low PI is less than 2.2, normal PI is 2.2–3.0 (reference value [20]) and high PI is greater than 3.0. PI was chosen over BMI as it may better reflect neonatal body proportionality, particularly in populations with variation in birth length [21].

Birth Weight Relative to Gestational Age and Sex: Assessed using the standardized birth weight z-score [22], divided into three categories: small for Gestational Age (SGA), where birth weight falls below the 10th percentile, corresponding to a z-score less than − 1.28 standard deviations; appropriate for gestational age (AGA), where birth weight ranges from the 10th to the 90th percentile, with a z-score from − 1.28 to 1.28 standard deviations (established as the reference group); and large for gestational age (LGA), where birth weight exceeds the 90th percentile, indicated by a z-score greater than 1.28 standard deviations.

Gestational age at delivery: Defined as the duration of a pregnancy, measured from the first day of the woman’s last menstrual period and is classified into three categories: preterm birth, which is less than 37 weeks of gestation [23]; term birth, which is between 37 and 41 weeks of gestation (serves as the reference category); and post-term birth, which is 42 weeks of gestation or more.

Season of birth: Categorized into four seasons based on date of birth: winter includes participants born in January, February, and March; spring includes participants born in April, May, and June; summer includes participants born in July, August, and September; and autumn includes participants born in October, November, and December.

### Outcome: Measures of BMD and BMC

BMD and BMC were measured for total hip and femoral neck (FN) using dual-energy X-ray absorptiometry (DXA). In HUNT3, all examinations were performed using one unit of the Lunar Prodigy Advance that was moved between the five major field stations. In HUNT4, this unit was used for two of the five field stations; otherwise, the measurements were performed at the two hospitals in Namsos and Levanger, using Lunar Prodigy Advance and the Hologic Horizon DXA systems, respectively. To account for the bone densitometry measurements in HUNT4 performed on two different machines, a conversion formula was applied for BMD, enabling the integration of data from both surveys [24]. For BMC, we included only measurements from the Lunar machine to ensure the accuracy and consistency of BMC measurements, as the conversion formula is not validated for BMC. Measurements from the left hip were preferred; however, when missing, measurements from the right hip were included (*N* = 19). We included the first bone measurement for all participants, using data from HUNT3 (*N* = 2,324 participants) when available, and from HUNT4 (*N* = 850) otherwise.

### Covariates

Potential confounders included sex, birth year, age at bone densitometry, maternal age, and maternal morbidity. Maternal morbidity was defined as the presence of selected health conditions recorded before or during pregnancy that may influence offspring bone outcomes. These data were obtained from MBRN [25], including chronic inflammatory joint disease, diabetes (both pre-existing and gestational), and pre-eclampsia/eclampsia. Each condition was included as a binary variable, coded as ‘yes’ or ‘no’, based on relevant diagnosis codes.

### Statistical Analysis

Descriptive data are presented as means with standard deviations (SD) for continuous data and numbers and percentages for categorical data. We used linear regression to estimate mean differences with 95% confidence intervals (CI) in BMD and BMC by birth characteristics, either estimated as differences between categories or per unit increase in continuous measures. Sex and maternal morbidity were included as categorical variables, while maternal age, age at bone densitometry, and birth year were continuous. In analyses of birth weight and ponderal index, we also adjusted for gestational age to isolate the specific effect of birth weight on the outcome variable*.* Residual analysis was conducted to evaluate the validity of the regression assumption.

We performed three sensitivity analyses: first, we restricted the analysis to HUNT participants randomly assigned to BMD measurements, excluding those who were recruited based on respiratory symptoms. Second, only participants who were examined using the same DXA machine (Lunar, specifically all from HUNT3 and a selection from HUNT4) were included, to avoid potential variability that could arise from using different machines (Lunar and Hologic) in HUNT4. Third, BMD at the total hip was analyzed to ensure consistent results across hip regions.

All statistical analyses were performed using Stata 18.0 (StataCorp LLC, College Station, TX, USA).

### Ethics

Participants in HUNT3 and HUNT4 gave written, informed consent for the data to be used for research, including a linkage with other registers. The study was approved by the Regional Committee for Medical Research Ethics, Central Norway (application number 246732) [15].

## Results

A total of 3,174 participants aged 20–54 years, with data on BMD and BMC from HUNT3 or HUNT4, were included. Among the participants, 59 (1.9%) were born with a low ponderal index, 123 (3.9%) had an LBW, 397 (12.5%) were classified as SGA, and 152 (4.8%) were born preterm (Table 1). The mean age of mothers at delivery was 26.0 years (SD 5.1). Preeclampsia/eclampsia was reported in 2.1% of all pregnancies, while maternal (pre-pregnancy or gestational diabetes), as well as chronic inflammatory joint disease, each affected 0.2% of pregnancies. At the time of the bone densitometry, the participants had a mean age of 34.2 years (SD 8.4), and the mean body mass index (BMI) was 26.4 kg/m^2^ (SD 4.8), with minor differences in BMI across birth weight categories. A total of 1,031 participants reported respiratory symptoms, use of medication, or history of asthma (Table 1).

**Table 1:** Characteristics of the participants, both at birth (1967–1997) and at baseline in HUNT 3 (2006–2008) or HUNT 4 (2017–2019), along with maternal information according to birth weight

Characteristics at birth	All	Birth weight, kg					
		< 2.5 kg	2.5–2.9 kg	3.0–3.4 kg	3.5–3.9 kg	4.0–4.4 kg	≥ 4.5 kg
No. of participants (%)	3,174	123 (3.9)	236 (7.4)	951 (30.0)	1,202 (37.9)	533 (16.8)	129 (4.1)
Preterm, <37 weeks	152 (4.8)	59 (48.0)	31 (13.1)	29 (3.0)	25 (2.1)	7 (1.3)	1 (0.8)
Term, 37–41 weeks	2,487 (78.4)	61 (49.6)	179 (75.8)	791 (83.2)	963 (80.1)	402 (75.4)	91 (70.5)
Post-term, ≥42 weeks	535 (16.9)	3 (2.4)	26 (11.0)	131 (13.8)	214 (17.8)	124 (23.3)	37 (28.7)
- Small for gestational age n (%) ^a^	397 (12.5)	89 (72.4)	159 (67.4)	149 (15.7)	–	–	–
- Appropriate for gestational age n (%) ^b^	2,492 (78.5)	32 (26.0)	76 (32.2)	797 (83.8)	1.178 (98.0)	404 (75.8)	5 (3.9)
- Large for gestational age n (%) ^c^	285 (9.0)	2 (1.6)	1 (0.4)	5 (0.5)	24 (2.0)	129 (24.2)	124 (96.1)
Maternal Characteristics							
Primiparous, n (%)	2,361 (74.4)	97 (78.9)	161 (68.2)	754 (79.3)	899 (74.8)	370 (69.4)	80 (62.0)
Maternal age years, mean (SD)	26.0 (5.1)	26.2 (5.7)	26.4 (5.5)	25.4 (4.8)	26.0 (5.1)	26.7 (5.2)	27.7 (5.4)
- ≤ 19, n (%)	239 (7.5)	8 (6.5)	16 (6.8)	88 (9.3)	85 (7.1)	37 (6.9)	5 (3.9)
- ≥ 35, n (%)	180 (5.7)	12 (9.8)	16 (6.8)	39 (4.1)	63 (5.2)	35 (6.6)	15 (11.6)
Pre-eclampsia/eclampsia, n (%)	65 (2.1)	11 (8.9)	5 (2.1)	15 (1.6)	15 (1.3)	14 (2.6)	5 (3.9)
Maternal diabetes, n (%) *	7 (0.2)	–	–	2 (0.2)	1 (0.1)	4 (0.8)	–
Rheumatic arthritis, n (%)	6 (0.2)	–	1 (0.4)	1 (0.1)	2(0.2)	2 (0.4)	-
Characteristics at HUNT (HUNT 3 or HUNT 4)							
Female, n (%)	1,900 (59.9)	78 (63.4)	158 (67.0)	621 (65.3)	698 (58.1)	294 (55.2)	51 (39.5)
No. of participants from HUNT3 (%)	2,324 (73,2)	97 (78.9)	166 (70.3)	696 (73.2)	899 (74,8)	363 (68.1)	103 (79.8)
Age, mean (SD) years	34.2 (8.4)	33.7 (8.8)	34.3 (8.2)	34.7 (8.6)	34.0 (8.2)	33.7 (8.6)	33.3 (7.5)
Height, mean (SD) cm	172.4 (9.0)	168.9 (9.3)	168.5 (8.5)	170.6 (8.8)	173.0 (8.4)	175.0 (8.9)	179.2 (8.8)
Weight, mean (SD) kg	78.6 (16.4)	77.6 (18.0)	74.1 (16.6)	76.3 (16.0)	78.9 (15.6)	82.1 (16.8)	86.6 (17.6)
BMI, mean (SD) kg/m^2^	26.4 (4.8)	27.2 (5.4)	26.0 (5.0)	26.3 (4.8)	26.3 (4.6)	26.9 (4.2)	26.4 (4.8)
Current smokers, n (%)	415 (13.3)	12 (10.1)	22 (15.0)	121 (12.9)	139 (11.8)	87 (16.7)	21 (16.5)
Lung symptoms **	1,031 (32.5)	53 (43.1)	84 (35.6)	321 (33.8)	368 (30.6)	155 (29.1)	50 (38.8)

^a^(*p* < 10), ^b^(*p* 10–90), ^c^(*p* > 90)¸

^*^Both pre-pregnancy and gestational diabetes

^**^Includes a wide range of respiratory symptoms, ever diagnosis of asthma or chronic obstructive pulmonary diseases, or use of asthma medication

Overall, ponderal index, birth weight, and gestational age were positively associated with BMD in young adults (Table 2). The mean BMD for all participants was 0.971 g/cm^2^ (0.953 g/cm^2^ for women and 0.998 g/cm^2^ for men). A standard deviation increase in ponderal index was associated with an increase of 0.024 g/cm^2^ (95% CI 0.006, 0.042) in BMD. For birth weight, the adjusted mean differences were 0.003 g/cm^2^ (95% CI 0.002, 0.004) for each 100 g increase and 0.015 g/cm^2^ (95% CI 0.009, 0.022) for each standard deviation increase, respectively.

**Table 2:** Association between birth characteristics and bone mineral density (BMD) among participants aged 20–54 years with bone densitometry in HUNT3 (2006–2008) and HUNT4 (2017–2019)

^Variables^	^N (%)^	^Mean BMD, g/cm2*^	^Crude mean difference BMD, g/cm2^	^Adjusted** mean difference BMD, g/cm2^	^95% CI (adjusted)**^
^Ponderal index^					
^ Continuous, weight (g) / length (cm)3^	^3,148 (100.0)^	^0.971^	^0.030^	^0.024^	^0.006 to 0.042^
^PI Categories:^					
^ < 2.2^	^59 (1.9)^	^0.945^	^−0.024^	^−0.030^	^−0.063 to 0.003^
^ 2.2–3.0^	^2,787 (88.5)^	^0.969^	^(reference)^	^(reference)^	^(reference)^
^ > 3.0^	^302 (9.6)^	^0.991^	^0.022^	^0.0143^	^−0.001 to 0.030^
^Birth weight^					
^ Continuous^ ^(per 100 g. increase)^	^3,174 (100.0)^	^0.971^	^0.003^	^0.003^	^0.002 to 0.004^
^ Continuous^ ^(per SD increase)^	^3,174 (100.0)^	^0.971^	^0.016^	^0.015^	^0.009 to 0.020^
^Birth weight category (kg)^					
^ < 2.5^	^123 (3.9)^	^0.951^	^− 0.0211^	^− 0.028^	^− 0.053 to − 0.003^
^ 2.5–2.9^	^236 (7.5)^	^0.954^	^− 0.0179^	^− 0.015^	^− 0.033 to 0.003^
^ 3.0–3.4^	^951 (30.0)^	^0.958^	^− 0.0140^	^− 0.009^	^− 0.020 to 0.002^
^ 3.5–3.9^	^1,199 (37.9)^	^0.972^	^(reference)^	^(reference)^	^(reference)^
^ 4.0–4.4^	^531 (16.8)^	^0.994^	^0.0218^	^0.022^	^0.008–0.0347^
^ ≥ 4.5^	^128 (4.0)^	^1.013^	^0.0410^	^0.031^	^0.007–0.054^
^Birth weight for gestational age and sex^					
^ Small for gestational age (SGA)^	^397 (12.5)^	^0.955^	^− 0.016^	^−0.012^	^− 0.026 to 0.001^
^ Appropriate for gestational age (AGA)^	^2,487 (78.5)^	^0.971^	^(reference)^	^(reference)^	^(reference)^
^ Large for gestational age (LGA)^	^284 (9.0)^	^0.996^	^0.026^	^0.023^	^0.007 to 0.039^
^Gestational length^					
^ Preterm,^ ^<37 weeks^	^152 (4.8)^	^0.966^	^− 0.008^	^− 0.014^	^− 0.039 to 0.011^
^ Term,^ ^37–41 weeks^	^2,543 (80.1)^	^0.974^	^(reference)^	^(reference)^	^(reference)^
^ Post-term,^ ^≥42 weeks^	^479 (15.1)^	^0.958^	^− 0.015^	^− 0.016^	^− 0.003 to − 0.001^

^*^The standard deviation (SD) of BMD in the sample was 0.135 g/cm^2^

^**^Adjusted for: Sex, birth year, age at BMD examination, maternal age and maternal morbidity. For ponderal index and birth weight we also adjusted for gestational length

When categorizing birth weight, individuals with a birth weight ≥ 4.5 kg had a 0.031 g/cm^2^ (95% CI 0.007, 0.054) higher mean BMD compared to those born with a birth weight between 3.5 and 3.9 kg. Similarly, individuals born LGA had a 0.023 g/cm^2^ (95% CI 0.007, 0.039) higher BMD compared to those born AGA. On the other hand, we found a 0.016 g/cm^2^ (95% CI − 0.003, − 0.001) lower BMD among those born post-term compared to those born at term.

When examining the association between birth characteristics and BMC at the femoral neck with Lunar (*N* = 2,755) (Table 3), the mean BMC for all participants was 5.398 g. A standard deviation increase in ponderal index and birth weight was associated with a 0.171 g (95% CI 0.048, 0.293) and a 0.146 g (95% CI 0.112, 0.181) higher BMC, respectively. Individuals born with birth weight below 2.5 kg and SGA had an adjusted mean difference of − 0.298 g (95% CI − 0.469, − 0.127) and − 0.142 g (95% CI − 0.235, − 0.049), respectively. On the other hand, individuals born in the highest birth weight category or LGA had an adjusted mean difference of 0.260 g (95% CI 0.103, 0.418) and 0.206 g (95% CI 0.098, 0.313), respectively.

**Table 3:** Association between birth characteristics and bone mineral content (BMC) among participants aged 20–54 years with bone densitometry in HUNT3 (2006–2008) and HUNT4 (2017–2019)*

^Variables^	^*N* (%)^	^Mean BMC, g**^	^Crude mean difference BMC, g^	^Adjusted** * mean difference BMC, g^	^95% CI^
^Ponderal Index (PI)^					
^ Continuous, weight (g) / length (cm)3^	^2,732 (100.0)^	^5.398^	^0.101^	^0.171^	^0.048 to 0.293^
^PI Categories^					
^ < 2.2^	^53 (1.9)^	^5.202^	^− 0.195^	^− 0.206^	^− 0.006 to 0.209^
^ 2.2–3.0^	^2,428 (88.9)^	^5.397^	^(reference)^	^(reference)^	^(reference)^
^ ≥ 3.0^	^251 (9.2)^	^5.457^	^0.061^	^0.102^	^− 0.006 to 0.209^
^Birth weight^					
^ Continuous^ ^(per 100 g. increase)^	^2,755 (100.0)^	^5.398^	^0.035^	^0.026^	^0.020 to 0.033^
^ Continuous^ ^(per SD increase)^	^2,755 (100.0)^	^5.398^	^0.196^	^0.146^	^0.112 to 0.181^
^Birth weight category (kg)^					
^ < 2.5^	^110 (4.0)^	^5.108^	^− 0.317^	^− 0.298^	^− 0.469 to − 0.127^
^ 2.5–2.9^	^206 (7.5)^	^5.140^	^− 0.285^	^− 0.200^	^− 0.324 to − 0.077^
^ 3.0–3.4^	^829 (30.1)^	^5.261^	^− 0.163^	^− 0.098^	^− 0.173 to − 0.024^
^ 3.5–3.9^	^1,042 (37.8)^	^5.425^	^(reference)^	^(reference)^	^(reference)^
^ 4.0–4.4^	^454 (16.5)^	^6.654^	^0.229^	^0.185^	^0.096 to 0.275^
^ ≥ 4.5^	^114 (4.1)^	^5.886^	^0.461^	^0.260^	^0.103 to 0.418^
^Birth weight for gestational age and sex^					
^ Small for gestational age (SGA)^	^342 (12.4)^	^5.227^	^− 0.175^	^− 0.142^	^− 0.235 to − 0.049^
^ Appropriate for gestational age (AGA)^	^2,167 (78.7)^	^5.402^	^(reference)^	^(reference)^	^(reference)^
^ Large for gestational age (LGA)^	^246 (8.9)^	^5,610^	^0.208^	^0.206^	^0.098 to 0.313^
^Gestational length^					
^ Preterm, <37 weeks^	^136 (4.9)^	^5.230^	^− 0.193^	^− 0.170^	^− 0.312 to − 0.028^
^ Term, 37–41 weeks^	^2,195 (79.7)^	^5.423^	^(reference)^	^(reference)^	^(reference)^
^ Post-term,^ ^≥42 weeks^	^424 (15.4)^	^5.325^	^− 0.089^	^− 0.024^	^− 0.105 to 0.058^

^*^For participants from HUNT3 and HUNT4 using the Lunar machine

^**^The standard deviation of BMC in the sample was 0.984 g

^***^Adjusted for: Sex, birth year, age at BMD examination, maternal age and maternal morbidity. For ponderal index and birth weight we also adjusted for gestational length

Among the birth characteristics examined, birth weight ≥ 4.5 kg showed the strongest positive association with both BMD and BMC. In contrast, birth weight < 2.5 kg, low ponderal index (< 2.2), and being small for gestational age (SGA) were associated with the lowest values of BMD and BMC.

No nominally statistically significant differences were found in femoral neck BMD across seasons. The BMD values were as follows: winter 0.973 (CI 0.620–1.561), spring 0.968 (CI 0.622–1.491), summer 0.973 (CI 0.642–1.410), or autumn 0.971 (CI 0.614–1.609).

### Sensitivity Analysis

In the sensitivity analysis excluding participants with respiratory symptoms (*N* = 1,031), we observed positive associations for the ponderal index and birth weight with BMD, consistent with the main analysis. Individuals born with lower birth weights and those classified as SGA had significantly lower BMD compared to their respective reference groups (Supplementary Table 1).

When including only participants examined with the same DXA machine (Lunar, *N* = 2,674), we found that higher PI and birth weights were associated with increased BMD. These results are also consistent with and support the findings of the main analysis (Supplementary Table 2).

Using BMD at the total hip instead of the femoral neck as the outcome also yielded similar results. Higher PI and birth weights were associated with increased BMD, further confirming the positive association between birth characteristics and BMD (Supplementary Table 3).

## Discussion

In this population-based cohort study, we found a positive association between the ponderal index, birth weight, birth weight for gestational age, and both BMD and BMC in young adults. These findings highlight the role of early life factors in influencing bone health.

Our results are consistent with previous research [4–12]. In two systematic literature reviews conducted in 2010 and 2011 [4, 5], most studies reported a positive association between birth weight and BMC. However, only two studies in the first review and one in the second review demonstrated an association between birth weight and adult BMD after adjustment for confounding factors. Since then, a few studies have reported an association between birth weight and BMD and BMC in adults. These studies primarily involved participants born with very low birth weight (< 1500 g) or very preterm (28–32 weeks of gestation), and extremely low birth weight (< 1000 g) or extremely preterm (< 28 weeks of gestation), compared to individuals with normal birth weight [6–9, 11, 12]. One study also compared preterm SGA-born individuals to term-born individuals [10]. In our study, only seven out of the 123 low birth weight participants (< 2500 g, LBW) were classified as having very low birth weight, with the average birth weight in the LBW group being 2,154 g. This highlights the importance of optimizing bone mass in all categories of LBW, not just those with very low or extremely LBW. Further, it is important to consider other factors that may influence BMD outcomes in these individuals. For example, research has shown that adults born with VLBW have poorer general, fine, and gross motor skills compared to those born at term [26] which may make them less likely to choose sports where these skills are important. Furthermore, they tend to exercise less during their leisure time than those born at term [27], which is unfavorable for optimizing peak bone mass [2]. This may also influence that these children spend less time outdoors, negatively affecting vitamin D synthesis and bone health.

When examining gestational length, we found that those born preterm had lower BMD compared to those born at term. For BMC, the findings were not statistically significant. Skeletal development takes place throughout the whole pregnancy, but the last trimester is particularly important, as it is estimated that 80 percent of mineral accretion in newborns occurs during this period [28, 29]. In preterm infants, it is likely that the reduced time in utero limits the period of bone mineralization, resulting in lower BMD and BMC at birth. On the other hand, participants born post-term also showed reduced BMD and BMC relative to term-born individuals. These findings suggest that both preterm and post-term birth may be associated with suboptimal bone development. The lower BMD observed in post-term infants may be due to complications associated with prolonged pregnancy, such as placental insufficiency, which can lead to impaired nutrient and oxygen supply during the extended gestation period [30]. Another possible explanation for our findings in post-term individuals is inaccurate determination of gestational age. Ultrasound has been shown to reduce the incidence of late and post-term pregnancies and the need for obstetric intervention [31], and became standard practice in Norway in antenatal care in the late 1980s and early 1990s [32]. Before this, gestational age was determined based on the last menstrual period, a method known to be inaccurate [33, 34]. Given that our cohort was born between 1967 and 1997, a significant proportion of gestational ages were likely determined by the last menstrual period rather than by ultrasound. Further, term can be misclassified in mothers with prolonged menstrual cycles. A 35-day cycle, for instance, would miscalculate the due date and extend the gestational length by seven days. Mothers with prolonged menstrual cycles may be more likely to have decreased BMD, a genetic trait they may pass on to their offspring [35].

Although the differences in BMD between birth weight categories may appear modest, they are consistent and significant. For example, participants born with birth weight ≥ 4.5 kg had a mean BMD 0.031 g/cm^2^ higher than the reference group, corresponding to 0.23 SD. Compared to those born < 2.5 kg, the difference was 0.059 g/cm^2^, or 0.44 SD. Given that a 1 SD increase in BMD is associated with a 50% reduction in fracture risk [36], these differences may be clinically meaningful.

We did not find any relationship between season of birth and BMD. Due to reduced sunlight at northern latitudes, the mothers may have lower maternal vitamin D levels in the winter months; however, vitamin D status of the mothers was not available [37, 38].

The main strength of this study is its large, population-based design with a long follow-up period and the ability for individual-level linkage with high-quality data from a mandatory national health registry. We had access to accurate, objectively measured birth characteristics in the MBRN and bone density measurements in HUNT.

The study has some limitations. The DXA measurements were performed on two different machines, which have been shown to produce significant differences [39]. To account for this, we used a conversion formula, and we performed sensitivity analyses including only measurements from Lunar, supporting our main findings. Furthermore, the MBRN did not record maternal smoking habits during pregnancy before 1999, which is significant because maternal smoking habits are a relevant confounder in this analysis. Additionally, selection bias may have contributed to the low number of LBW individuals in our study, as no information was available on whether individuals born LBW were less likely to participate in the HUNT survey. Furthermore, the study may lack power due to the small number of participants in some groups. In analyses of birth weight and ponderal index, we also adjusted for gestational age to isolate the specific impact of birth weight on the outcome variable. However, it is important to note that such adjustments may introduce bias [40]. Finally, a proportion of participants were included based on reported respiratory symptoms, a diagnosis of obstructive lung disease, or the use of asthma medication. Among these, many had asthma only in childhood. Sensitivity analyses excluding this group did not influence our estimates.

When examining associations between birth characteristics and later health outcomes, it is important to consider the different metrics. We included both birth weight as a continuous variable and birth weight relative to gestational age and sex to capture different aspects of fetal growth. Using birth weight relative to gestational age and sex allows for the identification of growth abnormalities such as SGA or LGA, which may reflect intrauterine growth restriction or overgrowth more accurately than birth weight alone. This approach provides a more nuanced understanding of fetal development and its potential long-term health implications [41].

Although we present both BMD and BMC, we have chosen to focus on BMD, which is used clinically both as a reference for osteoporosis and in the calculation of PBM [42]. Future research should explore whether the observed associations between birth characteristics and bone mineral density persist in older adulthood, and whether they are linked to increased fracture risk later in life.

## Conclusion

In this population-based cohort study examining the association between birth characteristics and bone mineral density and bone mineral content in young adults, we found that ponderal index, birth weight, and gestational age were associated with both BMD and BMC. These results highlight the importance of factors that influence bone health in early life.

## Supplementary Information

Below is the link to the electronic supplementary material.

Supplementary file1 (DOCX 20 KB)

Supplementary file2 (DOCX 20 KB)

Supplementary file3 (DOCX 19 KB)
