# Supplementary material for: Birth Characteristics and Bone Mineral Density and Content in Young Adults: The HUNT Study, Norway
Source: Calcif Tissue Int. 2025 Oct 22;116(1):130. doi: 10.1007/s00223-025-01441-2 (PMC12540558; doi:10.1007/s00223-025-01441-2)
Supplement: Supplementary file 1 — Supplementary file1 (DOCX 20 KB) [file 223_2025_1441_MOESM1_ESM.docx]

**Supplementary table 1.** Association between birth characteristics and bone mineral density (BMD) among participants in HUNT3 (2006-2008) and HUNT4 (2017-2019) without lung symptoms/disease

| ^Variables^ | ^N (%)^ | ^Mean BMD, g/cm²^ | ^Crude mean difference BMD, g/cm²^ | ^Adjusted* mean difference BMD, g/cm²^ | ^95 % CI^ |
| --- | --- | --- | --- | --- | --- |
| ^Ponderal Index^ | | | | | |
| ^Continuous, weight (g) / length (cm) 3^ | ^2,129 (100.0)^ | ^0.969^ | ^0.032^ | ^0.028^ | ^0.006 to 0.049^ |
| ^< 2.2^ | ^33 (1.6)^ | ^0.939^ | ^- 0.027^ | ^- 0.044^ | ^-0.089 to 0.001^ |
| ^2.2-3.0^ | ^1,876 (88.1)^ | ^0.966^ | ^(reference)^ | ^(reference)^ | ^(reference)^ |
| ^≥ 3.0^ | ^220 (10.3)^ | ^0.996^ | ^0.030^ | ^0.020^ | ^0.001 to 0.038^ |
| ^Birthweight category (kg)^ | | | | | |
| ^Continuous (per 100 g. increase)^ | ^2,143 (100.0)^ | ^0.971^ | ^0.002^ | ^0.002^ | ^0.001, 0.003^ |
| ^Continuous (per SD)^ | ^2,143 (100.0)^ | ^0.971^ | ^0.013^ | ^0.010^ | ^0.005, 0.016^ |
| ^< 2.5^ | ^70 (3.3)^ | ^0.957^ | ^-0.018^ | ^-0.024^ | ^-0.057 to 0.009^ |
| ^2.5-2.9^ | ^152 (7.1)^ | ^0.942^ | ^-0.003^ | ^-0.030^ | ^-0.053 to -0.007^ |
| ^3.0-3.4^ | ^630 (29.4)^ | ^0.956^ | ^-0.019^ | ^-0.011^ | ^-0.002 to 0.030^ |
| ^3.5-3.9^ | ^834 (38.9)^ | ^0.974^ | ^(reference)^ | ^(reference)^ | ^(reference)^ |
| ^4.0-4.4^ | ^378 (17.6)^ | ^0.984^ | ^0.010^ | ^0.014^ | ^-0.002 to 0.030^ |
| ^≥ 4.5^ | ^79 (3.7)^ | ^1.008^ | ^0.034^ | ^0.022^ | ^-0.008 to 0.052^ |
| ^Gestational group^ | | | | | |
| ^Small for gestational age (SGA)^ | ^257 (12.0)^ | ^0.945^ | ^-0.025^ | ^-0.020^ | ^-0.037 to -0.003^ |
| ^Appropriate for gestational age (AGA)^ | ^1,696 (79.1)^ | ^0.970^ | ^(reference)^ | ^(reference)^ | ^(reference)^ |
| ^Large for gestational age (LGA)^ | ^190 (8.9)^ | ^0.990^ | ^0.020^ | ^0.020^ | ^0.000 to 0.039^ |
| ^Gestational length^ | | | | | |
| ^Preterm, <37 weeks^ | ^98 (4.6)^ | ^0.975^ | ^0.004^ | ^0.006^ | ^-0.020 to 0.323^ |
| ^Term, 37-41 weeks^ | ^1,675 (78.2)^ | ^0.971^ | ^(reference)^ | ^(reference)^ | ^(reference)^ |
| ^Post term, ≥42 weeks^ | ^370 (17.3)^ | ^0.958^ | ^-0.012^ | ^-0.011^ | ^-0.025 to 0.004^ |

* Adjusted for: Sex, Birthyear, Age at BMD examination, Maternal age and maternal morbidity. For ponderal index and birthweight we also adjusted for gestational length.
