# Supplementary material for: Birth Characteristics and Bone Mineral Density and Content in Young Adults: The HUNT Study, Norway
Source: Calcif Tissue Int. 2025 Oct 22;116(1):130. doi: 10.1007/s00223-025-01441-2 (PMC12540558; doi:10.1007/s00223-025-01441-2)
Supplement: Supplementary file 2 — Supplementary file2 (DOCX 20 KB) [file 223_2025_1441_MOESM2_ESM.docx]

**Supplementary table 2.** Association between birth characteristics and BMD at femoral neck among participants from HUNT 3 (2006-2008) and HUNT4 (2017-2019) with Lunar DXA

| ^Variables^ | ^N (%)^ | ^Mean BMD, g/cm²^ | ^Crude mean difference BMD, g/cm²^ | ^Adjusted* mean difference BMD, g/cm²^ | ^95 % CI^ |
| --- | --- | --- | --- | --- | --- |
| ^Ponderal Index^ | | | | | |
| ^Continuous, weight (g) / length (cm) 3^ | ^2,653 (100.0)^ | ^1.033^ | ^0.030^ | ^0.024^ | ^0.004 to 0.044^ |
| ^< 2.2^ | ^50 (1.89)^ | ^1.002^ | ^-0.029^ | ^-0.034^ | ^-0.07 to 0.003^ |
| ^2.2-3.0^ | ^2,363 (89.1)^ | ^1.031^ | ^(reference)^ | ^(reference)^ | ^(reference)^ |
| ^≥ 3.0^ | ^240 (9.0)^ | ^1.054^ | ^0.022^ | ^0.014^ | ^-0.003 to 0.032^ |
| ^Birthweight category (kg)^ | | | | | |
| ^Continuous (per 100 g. increase)^ | ^2,674 (100.0)^ | ^1.033^ | ^0.003^ | ^0.003^ | ^0.002 to 0.004^ |
| ^Continuous (per SD)^ | ^2,674 (100.0)^ | ^1.033^ | ^0.016^ | ^0.015^ | ^0.009 to 0.021^ |
| ^< 2.5^ | ^106 (4.0)^ | ^1.015^ | ^-0.019^ | ^-0.025^ | ^-0.053 to 0.003^ |
| ^2.5-2.9^ | ^201 (7.5)^ | ^1.015^ | ^-0.018^ | ^-0.014^ | ^-0.035 to 0.006^ |
| ^3.0-3.4^ | ^800 (30.0)^ | ^1.018^ | ^-0.015^ | ^-0.011^ | ^-0.023 to 0.001^ |
| ^3.5-3.9^ | ^1,018 (38.0)^ | ^1.034^ | ^(reference)^ | ^(reference)^ | ^(reference)^ |
| ^4.0-4.4^ | ^444 (16.6)^ | ^1.061^ | ^0.028^ | ^0.025^ | ^0.011 to 0.040^ |
| ^≥ 4.5^ | ^111 (4.1)^ | ^1.069^ | ^0.035^ | ^0.026^ | ^0.001 to 0.052^ |
| ^Gestational group^ | | | | | |
| ^Small for gestational age (SGA)^ | ^332 (12.4)^ | ^1.023^ | ^-0.009^ | ^-0.006^ | ^-0.021 to 0.009^ |
| ^Appropriate for gestational age (AGA)^ | ^2,109 (78.7)^ | ^1.032^ | ^(reference)^ | ^(reference)^ | ^(reference)^ |
| ^Large for gestational age (LGA)^ | ^239 (8.9)^ | ^1.058^ | ^0.027^ | ^0.024^ | ^0.007 to 0.042^ |
| ^Gestational length^ | | | | | |
| ^Preterm, <37 weeks^ | ^130 (4.9)^ | ^1.022^ | ^-0.014^ | ^-0.012^ | ^-0.036 to 0.011^ |
| ^Term, 37-41 weeks^ | ^2,089 (78.0)^ | ^1.036^ | ^(reference)^ | ^(reference)^ | ^(reference)^ |
| ^Post term, ≥42 weeks^ | ^461 (17.2)^ | ^1.023^ | ^-0.013^ | ^-0.007^ | ^-0.021 to 0.006^ |

* Adjusted for: Sex, Birthyear, Age at BMD examination, Maternal age and maternal morbidity. For ponderal index and birthweight we also adjusted for gestational length.
