# Supplementary material for: Birth Characteristics and Bone Mineral Density and Content in Young Adults: The HUNT Study, Norway
Source: Calcif Tissue Int. 2025 Oct 22;116(1):130. doi: 10.1007/s00223-025-01441-2 (PMC12540558; doi:10.1007/s00223-025-01441-2)
Supplement: Supplementary file 3 — Supplementary file3 (DOCX 19 KB) [file 223_2025_1441_MOESM3_ESM.docx]

**Supplementary table 3.** Association between birth characteristics and bone mineral density (BMD) in total hip for participants in HUNT3 (2006-2008) and HUNT4(2017-2019)

| ^Variables^ | ^N (%)^ | ^Mean BMD, g/cm²^ | ^Crude mean difference BMD, g/cm²^ | ^Adjusted* mean difference BMD, g/cm²^ | ^95 % CI^ |
| --- | --- | --- | --- | --- | --- |
| ^Ponderal Index^ | | | | | |
| ^Continuous weight (g) / length (cm) 3^ | ^3,148 (100.0)^ | ^0.971^ | ^0.025^ | ^0.025^ | ^0.006 to 0.043^ |
| ^Ponderal Index categories^ | | | | | |
| ^< 2.2^ | ^59 (1.9)^ | ^0.945^ | ^0.017^ | ^0.020^ | ^-0.014 to 0.054^ |
| ^2.2-3.0^ | ^2,787 (88.5)^ | ^0.969^ |  |  |  |
| ^≥ 3.0^ | ^302 (9.6)^ | ^0.991^ | ^0.038^ | ^0.038^ | ^0.001 to 0.075^ |
| ^Birthweight category (kg)^ | | | | | |
| ^Continuous (per 100 g. increase)^ | ^3,174 (100.0)^ | ^0.971^ | ^0.002^ | ^0.002^ | ^0.001 to 0.003^ |
| ^Continuous (per SD)^ | ^3,174 (100.0)^ | ^0.971^ | ^0.013^ | ^0.010^ | ^0.005 to 0.016^ |
| ^< 2.5^ | ^123 (3.9)^ | ^0.951^ | ^-0.014^ | ^-0.017^ | ^-0.043 to 0.009^ |
| ^2.5-2.9^ | ^236 (7.5)^ | ^0.954^ | ^-0.015^ | ^-0.011^ | ^-0.030 to 0.008^ |
| ^3.0-3.4^ | ^951 (30.0)^ | ^0.958^ | ^-0.001^ | ^-0.004^ | ^-0.016 to 0.0007^ |
| ^3.5-3.9^ | ^1,199 (37.9)^ | ^0.972^ | ^0.000^ | ^0.000^ |  |
| ^4.0-4.4^ | ^531 (16.8)^ | ^0.994^ | ^0.018^ | ^0.016^ | ^0.002 to 0.029^ |
| ^≥ 4.5^ | ^128 (4.0)^ | ^1.013^ | ^0.036^ | ^0.024^ | ^0.000 to 0.048^ |
| ^Birth weight for gestational age and sex^ | | | | | |
| ^Small for gestational age (SGA)^ | ^397 (12.5)^ | ^0.955^ | ^-0.007^ | ^-0.005^ | ^-0.019 to 0.001^ |
| ^Appropriate for gestational age (AGA)^ | ^2,487 (78.5)^ | ^0.971^ | ^(reference)^ | ^(reference)^ | ^(reference)^ |
| ^Large for gestational age (LGA)^ | ^284 (9.0)^ | ^0.996^ | ^0.017^ | ^0.015^ | ^-0.001 to 0.031^ |
| ^Gestational length^ | | | | | |
| ^Preterm, <37 weeks^ | ^152 (4.8)^ | ^0.966^ | ^-0.005^ | ^-0.007^ | ^-0.028, 0.015^ |
| ^Term, 37-41 weeks^ | ^2,482 (78.4)^ | ^0.974^ | ^(reference)^ | ^(reference)^ | ^(reference)^ |
| ^Post term, ≥42 weeks^ | ^534 (16.8)^ | ^0.960^ | ^-0.015^ | ^-0.012^ | ^-0.024, 0.000^ |

* Adjusted for: Sex, Birthyear, Age at BMD examination, Maternal age and maternal morbidity. For ponderal index and birthweight we also adjusted for gestational length.
